# Animal Venoms for Drug Discovery: A Constantly Evolving Research Field

**DOI:** 10.3390/toxins18010030

**Published:** 2026-01-08

**Authors:** Ziad Fajloun, Rabih Roufayel, Mohamad Rima

**Affiliations:** 1 Department of Cell Culture, Laboratory of Applied Biotechnology (LBA3B), Azm Center for Research in Biotechnology and Its Applications, EDST, Lebanese University, Tripoli 1300, Lebanon; 2 Faculty of Sciences 3, Michel Slayman Tripoli Campus, Lebanese University, Ras Maska 1352, Lebanon; 3 College of Engineering and Technology, American University of the Middle East, Egaila 54200, Kuwait; rabih.roufayel@aum.edu.kw; 4 Department of Biological Sciences, Lebanese American University, Byblos P.O. Box 36, Lebanon

## 1. Introduction

Animal venoms, complex mixtures of molecules refined over thousands of years of evolution, represent far more than a simple defense or predatory system in venomous animals. These natural extracts constitute an exceptional chemical library for the design and discovery of new pharmacological agents of interest. The richness of animal venoms in peptides and proteins with remarkably specific and potent biological activities, acting through well-defined cellular and molecular mechanisms, makes them ideal candidates for addressing complex therapeutic targets, from neurological and cardiovascular diseases to cancers, including inflammatory and infectious disorders [1,2]. Historically, venom-based therapeutics have evolved considerably from early medicinal practices, in which snake and bee venoms were used in traditional cultures such as Egyptian, Greek, and Asian medicine for pain and inflammatory conditions. Modern drug discovery was able to translate this biological diversity into clinically approved agents [2,3], such as the angiotensin-converting enzyme (ACE) inhibitor captopril derived from *Bothrops jararaca* venom [4], the calcium channel blocker ziconotide from cone snail venom [5], and antiplatelet agents like eptifibatide and tirofiban from snake venom disintegrins [6]. Beyond their therapeutic potential, animal venoms have also been extensively studied for their toxicity and for the development of antivenoms to prevent lethal outcomes. Advances in biochemistry, proteomics, and genomics have enabled the identification and characterization of hundreds of peptides and proteins to be used as bioactive templates for drug design, or as immunogens for antivenoms production. These discoveries have paved the way for the use of animal venom-derived toxins as therapeutic models, inspiring the development of drugs capable of precisely targeting ion channels, receptors, or enzymes involved in human diseases [7].

The Special Issue of *Toxins*, entitled “Animal Venom in Drug Discovery: A Valuable Therapeutic Tool”, aimed to highlight cutting-edge research in the rapidly expanding field of biopharmaceutical applications of animal venoms. The studies gathered therein illustrate the diversity of approaches, animal sources, and therapeutic avenues being explored, demonstrating the vitality of this interdisciplinary field.

## 2. An Overview of Contemporary Venom Research

The scientific contributions in this Special Issue focused on several major themes, ranging from fundamental characterization of animal venoms to the precision engineering of venom-derived peptides.

### 2.1. Discovery and Characterization of New Bioactive Compounds

Several scientific studies highlight the valuable exploratory research aiming to understand animal venoms. The study by Wehbe et al. on the venom of the scorpion *Hottentotta judaicus* reveals a complex protein composition and demonstrates its novel neuromodulatory effects in invertebrate models, paving the way for the identification of new neurotoxins of interest [8]. Interestingly, this venom did not exhibit cytotoxic activity against most cancer cell lines, suggesting that its peptides may act selectively on neural cells, likely through interactions with ion channels. In agreement with this observation, a literature review published in this Special Issue provided a comprehensive analysis of scorpion venom peptides, focusing on their roles as voltage-gated sodium (Na_v_), potassium (K_v_), and calcium (Ca_v_) channel modulators [9]. By summarizing the latest progress in scorpion venom peptide research, this review discusses their mechanisms of action, therapeutic potential, and challenges in clinical translation. It complements the section and provides solid evidence that scorpion venom is a rich source of ion channel modulators worthy of further investigation [9]. Consistent with the ongoing discovery and exploration of animal venoms, the work of D’Amélio et al. on *Bothrops moojeni* venom highlights fractions capable of selectively modulating osteoclast function and differentiation. In fact, the venom differentially affects osteoclasts: its high-molecular-weight fraction disrupts cytoskeleton and bone resorption, while the low-molecular-weight (LMW) fraction selectively regulates osteoclast function, with minimal structural impact. By modulating cytokines and key signaling pathways, the LMW fraction emerges as a promising precision therapy for bone diseases with reduced side effects [10]. The immunomodulatory effects of animal venoms were further highlighted in the study by Ayoub et al., which investigated *Apis mellifera syriaca* venom. This venom modulated inflammatory cytokines (IL-1β, IL-6, TNF-α) in a hyperalgesia model, providing mechanistic evidence for its antinociceptive activity [11]. Taken together, these studies underscore that nature remains an essential source for the discovery of bioactive compounds, highlighting their unique mechanisms of action and therapeutic potential, and paving the way for the development of more selective and effective biomedical applications.

### 2.2. Exploration of New Therapeutic Applications

There is a growing effort to explore new therapeutic avenues based on animal-derived molecules, leveraging their unique bioactivities to address unmet clinical needs. The recent studies showcased in this Special Issue span the discovery of novel therapeutic applications of venom components such as the Vaa-snaclec-3/2 protein, derived from *Vipera ammodytes* venom. This C-type lectin-like protein prevents arterial thrombosis without inducing spontaneous hemorrhage or organ damage. These findings highlight this protein as a promising and safe candidate for transient thrombosis prevention in acute clinical settings [12]. Another study reported snake venom metalloproteinases as promising biotechnological tools for developing more effective treatments for toxoplasmosis, a major global health concern. These metalloproteinases can impair *Toxoplasma gondii* invasion and intracellular growth in human cells by modulating key cytokines involved in host susceptibility [13]. These findings underscore the strong potential of venom-derived compounds as promising scaffolds for the development of new agents targeting protozoan parasites. Other animal venom components, such as melittin from bee venom, phospholipase A_2_ from snake venom, and chlorotoxin from scorpion venom, also exhibit potent antimicrobial effects through mechanisms including membrane disruption, enzymatic inhibition, and immune modulation. These advances are thoroughly covered in a comprehensive review included in this Special Issue [14].

Another study investigated an elastase-inhibiting peptide extracted from centipede venom (ShSPI) in idiopathic pulmonary fibrosis (IPF), a chronic lung disease characterized by the fibrotic thickening of the alveolar walls. The peptide was able to significantly reduce bleomycin-induced pulmonary fibrosis in mice [15]. After inhalation, ShSPI improves the animal’s overall condition, attenuates lung damage, limits inflammatory cell infiltration, collagen deposition, and the formation of fibrous nodules. These effects are accompanied by a reduction in pro-inflammatory cytokines (IL-6, IL-1β, MCP-1) and an increase in anti-inflammatory IL-10 [15]. This study perfectly illustrates the remarkable therapeutic diversity of venoms, which extends far beyond their classical toxic effects on microbes and parasites to encompass complex beneficial mechanisms in multicellular organisms and disease contexts. These studies demonstrate that venoms represent an evolving reservoir of rare biomolecules with significant potential for modern pharmacology. Through synthetic production and peptide engineering, these molecules can be optimized to enhance their biological activities, opening new avenues for innovative treatments against fibrosis, infections, and cardiovascular diseases.

### 2.3. Engineering and Optimization of Venom-Derived Molecules

Engineering and optimizing previously discovered molecules are crucial steps in the development of new drugs and offer a powerful strategy to enhance their selectivity and biological activity, enabling the development of more effective therapeutics. The BF9 peptide, a trypsin-like serine protease inhibitor (Kunitz-type protein inhibitor) derived from the venom of *Bungarus fasciatus*, perfectly illustrates this approach. In fact, its analog BF9-N17K, obtained through targeted amino acid modifications, exhibits increased selectivity for coagulation factor XIa while reducing its activity on the homeostatic plasmin, paving the way for safer anticoagulants [16]. Since optimizing previously discovered molecules is not the only path to improving venom-derived therapeutics, several studies have focused on engineering venom-derived peptides and enhancing their large-scale production. This effort is critical for translating their therapeutic potential into clinically and commercially viable applications. In this context, Jararhagin-C (JarC), a *Bothrops jararaca* venom protein with disintegrin-like and cysteine-rich domains, was successfully produced in recombinant form (rJarC) using a bicistronic vector. The SUMO-fusion system enabled efficient expression and cleavage, yielding rJarC that retained the expected molecular mass and recognition by anti-jararhagin antibodies. Importantly, both native and recombinant JarC were non-toxic to HUVEC cells and effectively modulated cell migration, confirming the preservation of its biological activity [17]. This achievement of this study provides a feasible route for large-scale production and further therapeutic exploration of this venom-derived peptide.

These publications demonstrate that peptides derived from animal venoms uniquely combine high specificity with potent biological activity while remaining amenable to therapeutic modification. Through targeted engineering, these peptides and enzymes can be optimized to enhance efficacy and minimize toxicity. Snake venoms, for example, continue to inspire the development of novel treatments for thrombosis, infections, and other diseases. Their remarkable structural and functional diversity represents a rich reservoir for innovative drug discovery. Fully harnessing this potential, however, requires a deep understanding of their mechanisms and biological interactions, a task now accelerated by advances in biotechnology, proteomics, and molecular design. These approaches are already being applied, with patented snake venom-derived products highlighting their therapeutic potential for coagulation disorders, cancer, inflammation, and pain management [18].

## 3. Perspectives and Conclusions

The research presented in this Special Issue highlights a rapidly maturing field, revealing three major trends. First, there is a clear diversification of both sources (scorpions, centipedes, bees) and therapeutic targets (thrombosis, fibrosis, parasitic infections, pain, bone remodeling), extending far beyond the historical focus on neurotoxins and anticoagulants. Second, studies are moving from simple characterization toward precision engineering to improve the selectivity, activity, and production properties of candidate molecules. Third, multi-omics approaches and functional screening have become essential tools for deciphering venom complexity and identifying bioactive compounds. Despite these advances, challenges remain: much of the biodiversity of venomous animals is still understudied, translating preclinical discoveries into clinical applications demands sustained investment and innovative partnerships, and the sustainable, ethical production of venoms, along with alternative screening methods to reduce animal use, remains a critical concern for the future of the discipline. In addition, translational research in venom therapeutics is inherently complex: venoms contain hundreds of bioactive peptides and proteins whose composition varies with species and other factors, making isolation, characterization, and standardization challenging. Moreover, venom peptides often exhibit poor stability, low bioavailability, and potential immunogenicity, and preclinical models do not always reliably predict human outcomes, necessitating multidisciplinary strategies that extend well beyond the preclinical phase [2,19].
